# Investigation of viral etiology in potentially malignant disorders and oral squamous cell carcinomas in non-smoking, non-drinking patients

**DOI:** 10.1371/journal.pone.0232138

**Published:** 2020-04-29

**Authors:** Philippe Pérot, Michaël Falguieres, Laurence Arowas, Hélène Laude, Jean-Philippe Foy, Patrick Goudot, Nicole Corre-Catelin, Marie-Noëlle Ungeheuer, Valérie Caro, Isabelle Heard, Marc Eloit, Antoine Gessain, Chloé Bertolus, Nicolas Berthet

**Affiliations:** 1 Pathogen Discovery Laboratory, Institut Pasteur, Biology of Infection Unit, Paris, France; 2 Institut Pasteur, Centre national de référence (CNR) des papillomavirus humains (HPV), Paris, France; 3 Centre Léon Bérard, Centre de recherche en cancérologie de Lyon, Université de Lyon, Université Claude Bernard Lyon 1, Lyon, France; 4 Department of Oral and Maxillo-Facial Surgery, Pitié-Salpêtrière Hospital, Paris, France; 5 Institut Pasteur, Clinical Investigation and Acces to Bioresources Department, Paris, France; 6 Institut Pasteur, Unité Environnement et risques infectieux, Cellule d’Intervention Biologique d’Urgence, Paris, France; 7 National Veterinary School of Alfort, Paris-Est University, Maisons-Alfort, France; 8 Institut Pasteur, Unité d'Epidémiologie et Physiopathologie des Virus Oncogènes, CNRS UMR3569, Paris, France; 9 Sorbonne University, Paris, France; Queen Mary University of London, UNITED KINGDOM

## Abstract

Head and neck squamous cell carcinomas (HNSCC) are the seventh most frequent cancers. Among HNSCCs, oral squamous cell carcinomas (OSCCs) include several anatomical locations of the oral cavity, but exclude the oropharynx. The known risk factors for OSCCs are mainly alcohol consumption and tobacco use for at least 75–80% of cases. In addition to these risk factors, *Human papillomavirus* (HPV) types 16 and 18, classified as high-risk (HR) HPV genotypes, are considered as risk factors for oropharyngeal cancers, but their role in the development of OSCC remains unclear. We tested the hypothesis of viral etiology in a series of 68 well-characterized OSCCs and 14 potentially malignant disorders (PMD) in non-smoking, non-drinking (NSND) patients using broad-range, sensitive molecular methodologies. Deep-sequencing of the transcriptome did not reveal any vertebrate virus sequences other than HPV transcripts, detected in only one case. In contrast, HPV DNA was detected in 41.2% (28/68) and 35.7% (5/14) of OSCC and PMD cases, respectively. Importantly, 90.9% (30/33) of these belonged to the *Betapapillomavirus* genus, but no viral transcripts were detected. Finally, high-throughput sequencing revealed reads corresponding to transcripts of the *Trichomonas vaginalis* virus (TVV), which were confirmed by RT-PCR in two OSCCs. Our results strongly suggest that *Alphapapillomavirus* genotypes classified as HR are not involved in the development of OSCCs in NSND patients and that known oncogenic infectious agents are absent in these specific OSCCs. Any possible direct or indirect role of *Betapapillomavirus* genus members and TVV in OSCCs remains speculative and requires further investigation.

## Introduction

In 2015, head and neck squamous cell carcinomas (HNSCC), i.e. cancers of the mouth, nose, throat, larynx and sinuses, affected more than 5.5 million people worldwide and caused more than 379,000 deaths [1]. HNSCC is the seventh most frequent cancer and the ninth most frequent cause of death from cancer. Among these cancers, oral squamous cell carcinomas (OSCCs) include cancers of the inner mucosa of the lips, the cheeks and vestibule, the mobile part of the tongue, gums, the floor of the mouth, the intermaxillary region as well as the hard and anterior surfaces of the soft palate, but exclude the oropharynx (i.e. tonsils, base of the tongue, the posterior surface of the soft palate and the walls of the oropharynx) [2]. The estimated annual incidence worldwide of OSCC is approximately 300,000 cases with a mortality rate of about 145,000 [3]. However, the overall burden of this cancer varies across continents, with Asian countries contributing more than half of the cases (56.2%), and Africa showing the lowest percentage of cases at 5.7%. The percentages for Europe and America are 20.4 and 22.7%, respectively [3]. A subset of OSCCs appears on pre-existing lesions with a predisposition for malignant transformation, called “potentially malignant disorders” (PMDs) [4]. This group of oral lesions mainly includes leukoplakia, erythroplakia, lichen planus and some other relatively rare disorders. Among them, leukoplakia is the most common lesion, with an estimated prevalence of 0.5% worldwide [4] and an annual transformation rate of approximately 1% [5], albeit higher in Asian countries [6]. Finally, the presence of epithelial dysplasia is considered the most important indicator of malignant potential [5].

The known risk factors for OSCC are mainly alcohol consumption, tobacco use and chewing betel for at least 75–80% of cases [7–9]. Although the first two main factors are independently associated, they also act synergistically [10]. Moreover, some studies confirm that 10–20% patients suffering from OSCC can be considered as non-smokers and non-drinkers (NSND) [11, 12]. In addition to these risk factors, the *Human papillomavirus* (HPV) types 16 and 18, classified as high-risk (HR) HPV genotypes, are also considered as risk factors for oropharyngeal cancer, but their role in the development of oral cavity cancer remains unclear [13]. The possibility that HPV may play a role in OSCC was first raised in 1983 [14]; since then, several studies have indicated the presence of HR-HPV DNA in a certain proportion of neoplasias, suggesting that some of them were virally induced cancers. However, for OSCCs, these first results were controversial due to highly variable prevalence rates, ranging from 17 to 85% [15]. The first meta-analysis in 2005 on 60 studies showed that HPV prevalence was higher in oropharyngeal SCCs (OPSCCs) (35.6%) than in OSCCs (23.5%) or laryngeal SCCs (LSCC) (24%) [16]. More recently, another meta-analysis, which included more than 50 studies between 2007 and 2017, showed that the prevalence of HPV was 24.4% in OSCCs with a strong disparity across geographical areas, and the highest prevalence in Asian countries (33.77% of cases) [17]. However, in a number of these studies, no specific information on the anatomical sub-localization of SCC in the tongue (mobile part *versus* base of the tongue) was available, which may lead to confusion between OPSCCs and OSCCs, and thus an overestimation of the true burden of HPV in OSCCs [18]. In OSCCs, HPV type 16 is the most commonly found genotype followed by types 18, 31 and 33 and a few others [16, 19–22]. The tongue and floor of the mouth are the anatomical locations of the tumors most often found infected with these above-mentioned HR-HPV genotypes [19, 20, 22, 23]. Moreover, histological and epidemiological studies have demonstrated significant trends for decreased tumor differentiation of oral cavity and oropharyngeal carcinomas over the last 30 years [24]. Therefore, the increase in undifferentiated cancers suggests that non-identified infectious agents may be involved in malignant disorders and OSCCs in NSND patients [24–27]. To date, a few studies have already specifically investigated an infectious cause in these NSND patients. For instance, Laco *et al*. (2010) detected HR-HPV DNA via PCR in only 12.5% of OSCC cases in patients who had no history of smoking or alcohol abuse, whereas Li *et al*. (2015) did not identify any oncogenic virus in a cohort of 20 OSCCs of the tongue in NS patients [28, 29].

The aim of this study was to test the hypothesis of a viral etiology due to a known viral agent such as HPV or another, unknown or unforeseen, agent associated with an oncogenic process in a series of well-characterized PMDs and OSCCs in NSND patients using very broad and sensitive methodologies. Firstly, HPV was investigated using two different methods. The first one consisted of the use of a DNA microarray (PapilloCheck HPV genotyping kit) that detects 24 genotypes belonging to the *Alphapapilliomavirus* genus and the second one was based on several PCR systems using consensus HPV primers, followed by amplicon sequencing and analysis of the sequences obtained. Secondly, a broad-spectrum transcriptomic approach (i.e. high-throughput sequencing (HTS) of total RNA), was used to explore other etiological causes. This latter methodology was developed to detect and characterize unknown or variant replicating viruses associated with several human diseases, especially cancers.

## Methods and materials

### PMD and OSCC samples

At the maxillofacial surgery department at the Pitié-Salpêtrière Hospital (Paris, France), NSND patients with PMD or OSCC were selected for our prospective study according to strict criteria between 2012 and 2015. Patients were included in the study if they were over 18 years of age and were considered NSND following commonly accepted criteria. In addition, we will include in this category all patients whose rare, or very rare, occasional use of alcohol or tobacco suggested that the development of their OSCCs were not related to these two risk factors. Consequently, the patients included in our study were (i) strictly NS or with a consumption equal to or less than 5 packs per year or having stopped smoking at least 15 years prior, regardless of the amount smoked and (ii) strictly ND or with a moderate consumption of 10 or 20 g of alcohol per day or less, with the majority of the cases being less than 1–2 drinks per month for women and men, respectively, or having stopped drinking at least 15 years prior, regardless of initial consumption. The only non-inclusion criteria were previous antecedents of oral cancer. The first criteria for inclusion of PMDs was the clinical diagnosis of leukoplakia. All other PMDs, such as erythroplakia, lichen planus, proliferative verrucous leukoplakia (PVL), etc were excluded. All clinical data related to the patient (age, sex and clinical history) or to his/her PMD or OSCC (anatomical location, malignant tumor classification (TNM) and histological criteria) were compiled in a database managed using the Voozanoo platform (http://www2.voozanoo.net/) on the ICAReB platform at the Institut Pasteur. All samples of patients included in the study were collected during their surgical treatment according to current cancer reference standards. Each investigated tissue sample was harvested from the main tumor removed during surgery. To preserve the nucleic acids as much as possible, the tissues were immediately frozen in dry ice in the operating room and then sent to the Institut Pasteur for long-term storage and molecular investigations. All the bio-resources were managed by the ICAReB platform, comprising RNA/DNA extraction, sample transport, registration and coding with regard to ethical compliance. In parallel to the molecular investigations, the entire tumor was analyzed by anatomical pathologists to determine the histological grade, the margins of the tumor and the two main histological criteria of its severity (perineural invasion and vascular embolus).

### DNA extraction and genotyping of HPV using PapilloCheck and PCRs

Frozen tumor tissues samples were stored at -80°C until total genomic DNA extraction using the DNeasy Blood and Tissue kit (Qiagen, CA, USA) according to the manufacturer’s instructions. Extracted DNA was quantified using the Qubit^®^ dsDNA BR Assay kit with the Qubit 2.0 fluorimeter (Life Technologies, CA, USA) and stored at -20°C until molecular investigations such as HPV genotyping. Firstly, this extracted DNA was investigated using the PapilloCheck HPV genotyping kit (Greiner BioOne, Frickenhausen, Germany) according to the manufacturer’s recommendations. This assay first included PCR amplification with fluorescent primers (CY5 fluorophore) specific to the HPV E1 gene and the cellular housekeeping gene ADAT1. The amplification products were then hybridized to a DNA microarray containing sequences for 18 HR genotypes (HPV16, 18, 31, 33, 35, 39, 45, 51, 52, 53, 56, 58, 59, 66, 68, 70, 73, 82) and six low-risk (LR) genotypes (HPV6, 11, 40, 42, 43 and 44/55). Secondly, three consensus PCR assays were performed using three sets of degenerate primers (GP5+/6+, CP65-70 and CP66-69) [30, 31]. The first reaction using GP5+/6+ primers was performed in 100 μL using 5 μL of template DNA, 1X PCR buffer (Thermo Fisher Scientific), 3.5 mM MgCl_2_, 0.2 μM of each deoxynucleotide triphosphate (dNTP), 0.5 μM of each consensus primer GP5+/6+ and 1 U of Ampli Taq Gold DNA polymerase (Thermo Fisher Scientific). The second nested PCR assay was also performed using two sets of degenerate primers (CP65-70 and CP66-69). The reaction was performed in 50 μL including 5 μL of template DNA (either template of DNA or amplicon from PCR using the CP67-70 primers), 1X PCR buffer (Thermo Fisher Scientific), 3.5 mM MgCl_2_, 0.2 μM of each dNTP, 0.5 μM of each consensus primer and 1 U of Ampli Taq Gold DNA polymerase. Thermal cycling for the three PCRs are described in S1 Table. All PCR products were sequenced using the BigDye Terminator v3.1 Cycle Sequencing kit (Applied Biosystems) according to the manufacturer’s protocol.

### Viral metatranscriptomics

Total RNA was extracted from biopsies using the Trizol procedure (Thermo Fischer Scientific), purified on RNeasy column including a DNase treatment (Qiagen), and randomly amplified [32] for each patient individually. High-molecular-weight DNA was used for library construction and high-throughput sequencing (HTS) was subcontracted to DNA Vision (Charleroi, Belgium) on a HiSeq 2000 instrument, generating an average of 92 million reads per sample (min = 46 M; max = 162 M). Sequences were trimmed and then screened for viral sequences using the protein version of RVDB database [33]. Additional screening for bacteria and other non-viral microorganism sequences was performed using Centrifuge [34] and Metaphlan2 [35]. The reads and contigs of interest were mapped using the Geneious mapper of Geneious 11.1.5 [36] with the medium sensitivity/fast parameters.

### RT-PCR for detection of HR-HPV and the *Trichomonas vaginalis* virus

Reverse transcription reactions were performed starting from RNA with the SuperScript III First-Strand Synthesis System (Thermo Fischer Scientific). Then, qPCRs were performed in SYBR Green format with 45 cycles of amplification using primers described in S1 Table. Human beta actin was used as a positive internal control with primers ACTB-fw and ACTB-rv (S2 Table). Primers for the *Trichomonas vaginalis* virus 1, 2, 3 and 4 (TVV1, 2, 3, 4) were retrieved from [37]. Additional PCR systems for TVV1, 2 and 3 were designed from NGS reads (S2 Table). Finally, primers used for the detection of *T*. *vaginalis* (TV) were retrieved from [38] and designed according to conserved region of TV cysteine proteinase 4 (S2 Table).

### Ethical considerations

This study was approved by both the French Health Products Safety Agency (then called AFSSAPS, then ANSM since May 2012) on 20 February 2012 (B120225-40) and by the Ile de France VI Ethics Committee on 2 March 2012 (CPP no. 13–12 –IDRCB 2012-A00116-37). Written informed consent was obtained from all patients included in this study.

## Results

### Description of PMD and OSCC samples

This study included 14 PMD and 68 OSCC cases. The sex ratio (M:F) was 0.16 and 0.94 with an average age of 70 years (+/- 15 years) and 69 years (+/- 15 years) for PMDs and OSCCs, respectively (Table 1), with 86.7% of patients being over 50 years of age in this study. Given that the main inclusion criteria for patients was based on their alcohol and tobacco consumption, 42.8% (6/14) and 45.6% (31/68) of PMD and OSCC patients, respectively, had never been drinkers nor smokers. Moreover, for PMDs, 100% of these cases were observed in females and, for OSCCs, 22.6% (7/31) and 77.4% (24/31) were observed in males and females, respectively. The others were either moderate alcohol and/or tobacco users or stopped drinking or smoking more than 15 years ago. In 78.6% of cases (11/14), PMDs were located on the mobile part of the tongue, with a possible extension to other anatomical locations. On the other hand, 75% (51/68) of the OSCCs were found either in the mobile part of the tongue or in the gums (Table 1). Cheeks were the third identified anatomical location of OSCCs with 10.3% (7/68) and the other localizations represented less than 10% of cases. Histological results of PMD samples showed that they could be separated into simple hyperplasias (n = 3), dysplasias of various grades (I to III) (n = 7), and *in situ* carcinomas (n = 6), which represented 42.8% (6/14) of cases. In OSCC samples, histological criteria classified lesions according to the level of cell differentiation and keratinization. More than 83.9% (47/56) of OSCCs were keratinized, but data on this criterion could not be obtained for 17.6% (12/68) of cases. In parallel, in 35.3% and 55.9% of cases (24/68 and 38/68), the cells were moderately or well differentiated, respectively. Poorly differentiated cells were found in only 7.3% of cases. Finally, a history of PMD was found in 17.6% (12/68) of the OSCC cases in this study (S5 Table). They had the same anatomical location in 75% of cases (9/12). Moreover, depending on the case, these PMDs appeared to have developed over a period ranging from 1 year to about 20 years (S5 Table).

**Table 1:** Summary of clinical and pathological data on the potentially malignant disorder (PMD) and oral squamous cell carcinoma (OSCC) cases included in this study.

		PMDs			OSCCs		
		Overall	Female	Male	Overall	Female	Male
**Number of cases**		14	12	2	68	35	33
**Average age (years)**		70	73	50	69	72	65
**Age (years)**	Range	34–84	51–84	34–67	29–91	35–91	29–88
	< 30	0	0	0	1	0	1
	30–50	1	0	1	8	3	5
	50–70	5	4	1	26	14	12
	70 +	8	8		33	18	15
**Number of patients who have never drunk or smoked**		6	6	0	31	24	7
**Anatomical location**	Inner mucosa of lips	1	1	0	1	1	0
	Mobile part of the tongue	11	9	2	25	12	13
	Floor of the mouth				1	0	1
	Gum	1	1	0	26	16	10
	Cheek mucosa	1	1	0	7	2	5
	Intermaxillary region				4	1	3
	Hard palate of the mouth				3	2	1
	Soft palate of the mouth				1	1	0
	Hyperplasia	3	3	0	Not applicable		
	Dysplasia Grade I	4	3	1			
	Dysplasia Grade II	2	2	0			
	Dysplasia Grade III	1	1	0			
	*In situ* Carcinoma	6	5	1			
**Cellular differentiation**	Well	Not applicable			38	17	21
	Medium				24	14	10
	Poor				5	3	2
**Keratinization**	Yes	Not applicable			47	23	24
	No				9	8	1
	Not Determined				12	5	7
**Perineural invasion**	Yes	Not applicable			26	14	12
	No				38	18	20
	Not applicable				4		
**Vascular embolus**	Yes	Not applicable			13	9	4
	No				55	23	29
	Not applicable				3		

### Search of HPV DNA using conventional methods

At least one HPV type was detected in 35.7% (5/14) and 41.2% (28/68) of PMD and OSCC cases, respectively (See S3 & S4 Tables for results by molecular methods for HPV detection). Of these, 91.2% (31/34) belonged to the *Betapapillomavirus* genus (β-HPVs) (Table 2, S5 & S6 Tables). Only three HPV genotypes classified as high-risk (HPV16 and HPV33) were identified in three OSCC samples (CE03, CE07 & CE41). Two samples with HPV16 DNA were found in women aged 49 and 61 years, and HPV33 DNA was identified in a 53-year-old male. These three OSCC cases with HR-HPV were located in the mobile part of the tongue and histological analyses showed good differentiation and keratinization. These three HR-HPV accounted for either 4.4% of all OSCC cases or 12% of cases in the mobile part of the tongue. However, for only one of these cases (HPV16, in sample CE41), a PMD lesion had previously been reported at the same anatomical location, with development over less than one year (S6 Table). All the other β-HPVs identified in the OSCCs came from 69.2% (18/26) of cases involving either the mobile part of the tongue or the gum (Table 2). Among these OSCCs in which β-HPV was detected, the cells were moderately or well-differentiated only in 88.5% (23/26) of cases and keratinized in only 84.2% (16/19) of cases (S6 Table). Finally, in 92.3% (24/26) of these cases, no previous PMDs were indicated. In two cases, a PMD had been reported in the same anatomical location (S6 Table). These β-HPVs were also identified in 35.7% (5/14) of PMD cases. All β-HPVs were identified only in PMDs located at the tip or lateral part of the tongue (S5 Table). Moreover, they were found in equivalent proportions as simple hyperplasias or grade I or II (3/5) or *in situ* carcinomas (2/5). Overall, 15 different genotypes of β-HPV were identified in PMDs and OSCCs. However, approximately 86.7% (13/15) of these genotypes were found only once or twice, such as the HPV9 genotype, which was detected only in one PMD case (PM01). Finally, the HPV20 and HPV36 genotypes represented 46.2% (12/26) of the β -HPVs identified in both PMDs and OSCCs.

**Table 2:** Summary of the number of *Human papillomavirus* (HPV) genotypes detected according to anatomical location of the potentially malignant disorders (PMD) and oral squamous cell carcinomas (OSCC) included in this study.

			Anatomical localization			
		Overall	Mobile part of the tongue	Gum	Cheek	Others
**PMD**	**All HPVs**	5	5			
	**Alphapapillomavirus HPV**	0	0			
	**Betapapillomavirus HPV**	5	5			
**OSCC**	**All HPVs**	29	13*	8	5	3
	**Alphapapillomavirus HPV**	3	3	0	0	0
	**Betapapillomavirus HPV**	26	10	8	5	3

* one co-infection with α-& β-HPVs

### Viral metatranscriptomics

**Fig 1:**
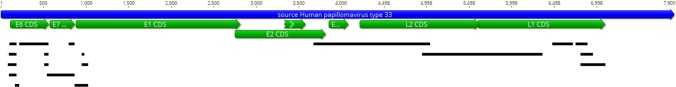
Mapping of singletons and contigs generated after *de novo* assembly of reads from sample CE07 on a reference sequence of the *Human papillomavirus* type 33 (HPV33) genome.

**Fig 2:**
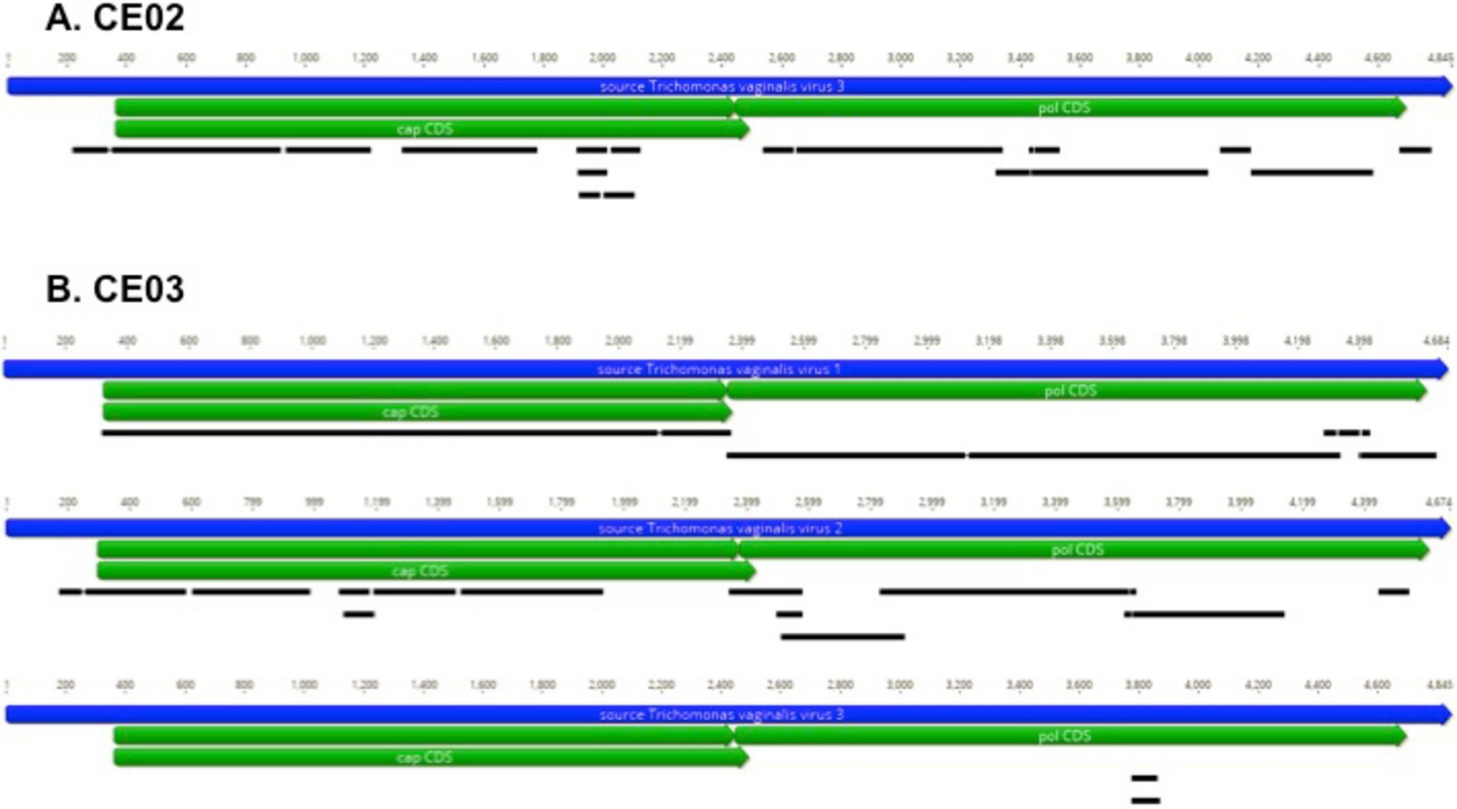
Mapping of the singletons and contigs generated after *de novo* assembly of the reads obtained for samples CE02 (A) and CE03 (B) on the reference sequences for *Trichomonas vaginalis* virus type 1, 2 and 3 genomes.

A viral etiological association was investigated using a metatranscriptomics agnostic approach based on the detection of all viral RNAs, in 7 and 19 PMD and OSCC cases, respectively. Most of the PMDs investigated came from the mobile part of the tongue (4/7), but the others came from either the lip, gum or cheek mucosa. Of these, only three β-HPVs had previously been identified using conventional DNA PCR. With the exception of the detection of bacterial transcripts most likely coming from commensal flora (data not shown), these analyses did not reveal any transcripts corresponding to β-HPV or belonging to other viral agents (Table 3). In the OSCCs, 84% of the samples investigated using HTS came mainly from the mobile part of the tongue (9/19) and the gum (8/19). In contrast to the results obtained for PMDs, transcripts corresponding to HPV33 were found in the CE07 sample in which this genotype had previously been detected using PCR (Table 3). These transcripts cover the entire genome with the exception of two viral genes E1 and E2 (Fig 1). However, similarly to PMDs, no other transcripts corresponding either to HPV16 (CE03) or to β-HPVs were detected using HTS, despite a high sequencing depth for the CE03 sample (about 150 million reads; see Table 3). In contrast, in two OSCCs (CE02 and CE03), thousands of unique reads corresponding to the *T*. *vaginalis* virus (TVV), a double-stranded RNA virus, belonging to the *Totiviridae* family, were identified. The analyses showed that these reads corresponded to the TVV1-2-3 and TVV3 viruses in CE03 and CE02, respectively (Fig 2 & Table 3). The presence of these TVV genotypes in these two OSCCs was confirmed either using RT-PCRs from the literature or with specific primers designed from the sequences obtained (Table 4). Moreover, the presence of the parasite itself (TV) was also demonstrated in these two samples by the identification of reads corresponding to specific housekeeping genes, including those of actin and phosphoenolpyruvate carboxykinase (data not shown). However, the presence of TV could not be directly confirmed using a reference RT-PCR [38] (Table 4).

**Table 3:** Compilation of the results from the analysis of metagenomic data and RT-PCR obtained for the oral squamous cell carcinoma (OSCC) and potentially malignant disorder (PMD) cases analyzed using high-throughput sequencing (HTS).

	Anatomical location	Number of samples	Number of raw HTS reads (10^6)	HTS results (viral hits) (2019)	RT-PCR ACTB	RT-PCR HPV33	RT-PCR HPV16 E6E7
PMD	Mobile part of the tongue	PM 01	79.9	Neg	**Pos**	Neg	Neg
		PM 04	96.0	Neg	**Pos**	Neg	Neg
		PM 05	97.6	Neg	**Pos**	Neg	Neg
		PM 06	83.6	Neg	**Pos**	Neg	Neg
	Cheek mucosa	PM 02	105.4	Neg	**Pos**	Neg	Neg
	Gum	PM 03	53.2	Neg	**Pos**	Neg	Neg
	Inner mucosa of lips	PM 07	82.8	Neg	**Pos**	Neg	Neg
OSCC	Gum	CE 01	79.8	Neg	**Pos**	Neg	Neg
		CE 02	83.4	TVV3	**Pos**	Neg	Neg
		CE 09	66.8	Neg	**Pos**	Neg	Neg
		CE 13	87.7	Neg	**Pos**	Neg	Neg
		CE 14	97.7	Neg	**Pos**	Neg	Neg
		CE 17	147.6	Neg	**Pos**	Neg	Neg
		CE 26	162.0	Neg	**Pos**	Neg	Neg
		CE 29	57.8	Neg	**Pos**	Neg	Neg
	Intermaxillary region	CE 15	107.7	Neg	**Pos**	Neg	Neg
	Mobile part of the tongue	CE 03	147.1	TVV1-2-3	**Pos**	Neg	Neg
		CE 04	83.3	Neg	**Pos**	Neg	Neg
		CE 07	97.2	HPV33	**Pos**	**Pos**	Neg
		CE 10	112.0	Neg	**Pos**	Neg	Neg
		CE 11	87.7	Neg	**Pos**	Neg	Neg
		CE 20	60.4	Neg	**Pos**	Neg	Neg
		CE 24	114.6	Neg	**Pos**	Neg	Neg
		CE 37	46.0	Neg	**Pos**	Neg	Neg
	Cheek mucosa	CE 06	57.0	Neg	**Pos**	Neg	Neg
		CE12	112.2	Neg	**Pos**	Neg	Neg

**Table 4:** Detection of *Trichomonas vaginalis* (TV) and the TV virus (TVV) using RT-PCR in oral squamous cell carcinoma (OSCC) cases.

		*Trichomonas vaginalis*	TVV1			TVV2			TVV3			TVV4
		TV-CP4-920***	TVV1-569**	TVV1-810-199*	TVV1-3036-194*	TVV2-20778-199*	TVV2-20778-202*	TVV2-625**	TVV3-440**	TVV3-4640-193*	TVV3-4640-202*	TVV4-514**
Gum	CE 02	Neg	Neg	Neg	Neg	Neg	Neg	Neg	Neg	**Pos**	**Pos**	Neg
Mobile part of the tongue	CE 03	Neg	Neg	**Pos**	**Pos**	**Pos**	**Pos**	**Pos**	**Pos**	**Pos**	Neg	Neg

*designed based on sequence data obtained from high-throughput sequencing

** from [37]

*** from [38]

## Discussion

In this study, a search of an association with an infectious agent was investigated using various molecular methods in a well-characterized series of PMD and OSCC in NSND patients. The epidemiology of these OSCCs is different from those with known risk factors (alcohol and tobacco). First, these alcohol and tobacco related cancers are generally more common in 60-year-old men and are located mainly in the tongue (25%), the floor of the mouth (17%) and the gums (13%) (unpublished data), whereas our NSND cohort was dominated by women over 70 years of age. Moreover, these cancers in NSND patients were mainly located in the mobile part of the tongue and gums, which represented more than 70% of the cases, with localization in the floor of the mouth being rare. These data confirm that cancers in NSND and SD are epidemiologically different. Therefore, in the absence of the two main known risk factors (i.e. tobacco and alcohol use), we investigated HR-HPV as an infectious etiological cause in our NSND cohort. However, our results differ from previous studies because we focused strictly on lesions localized in the oral cavity in NSND patients. Our investigations revealed HR-HPV DNA in only 4.4% of OSCCs in our cohort. Consequently, our study shows that the potential impact of HR-HPV in NSND patients as an etiological agent is lower compared with the majority of other studies in which the OSCCs studied also included patients with the two main risk factors (alcohol and/or tobacco consumption) [16, 19–22]. Smoking, rather than high-risk sexual behavior or immunodeficiency related to HIV infection, can increase the risk of HR-HPV infection in the oral cavity [23, 39, 40]. However, several studies have shown that the expression of the HPV E6/E7 genes occurs in only 6–7% of cases, confirming that the detection of HPV DNA must be distinguished from its oncogenic activity and that HPV detected may not be biologically active in the majority of OSCCs [20, 41, 42]. In our study, although the DNA of three HR-HPVs was detected, viral transcripts were found only in one case in which the patient was immunodeficient due to HIV infection (HPV33-CE07). No transcript of the HPV16 genotype was detected either using HTS or an RT-PCR specific to this genotype in the other two HR-HPV cases (CE03 and CE41). In conclusion, all our data suggest that the impact of HR-HPV in NSND patients is even lower than previously estimated, because the only OSCC case possibly driven by HR-HPV was identified in a patient co-infected with HIV. These data raise the question of whether OSCCs in which HR-HPV DNA are detected are really HPV-driven, given that the presence of HPV does not mean that it is biologically active.

Nonetheless, this study highlighted the presence of a β-HPV in 35.7% and 41.2% of cases, for PMDs and OSCCs, respectively. Many studies mention that β-HPVs are ubiquitously disseminated throughout the human body, participating in commensal flora (even though β-1 and β-2 HPVs are more prevalent in cutaneous areas whereas β-3 is more abundant in the nasal cavity than on the skin [43, 44]). In addition, these β-HPVs are more often found in keratinized tissues, such as the mobile part of the tongue and gums than in the cheek or the floor of the mouth, which are free of keratin [45]. Some of these cutaneous HPVs are associated with a wide variety of clinical manifestations in the skin [46]. However, the etiological role of these β-HPV in skin cancers is difficult to prove given the high heterogeneity that exists within this group of viruses and that they are found ubiquitously at many anatomical sites [47, 48]. The only evidence of the association between β-1 HPV and skin lesions with carcinogenicity is in epidermodysplasia verruciformis (EV), a rare autosomal recessive hereditary skin disease, in which genotypes HPV5 and HPV8, but also HPV14, HPV20 and some others have been found in these lesions [49]. In addition, viral transcripts and a high number of viral genomes are generally found in EV [50]. However, unlike in EV, for the other β-HPVs detected in various other SCCs of the skin, the viral load is generally less than one copy per cell [51] and no transcript of these papillomaviruses can be identified [52] showing that β-HPVs may not be expressed in these other skin SCCs. Unlike α-HPVs associated with cancers that are transcriptionally active and are absolutely required for cancer maintenance, the role of β-HPVs may be relatively more important for the initiation of cancer than in its maintenance. The E6 protein of β-HPV can target many pathways or proteins such as p300, MAML1 or Notch, which in particular has tumor suppressor gene functions in epithelial cells [53–55]. The effect of E6's interaction with these cellular targets may allow HPV-infected cells to accumulate many mutations that would be necessary and then sufficient for the cell to progress to cancer. However, since these β-HPVs are also found in other compartments, the association with SCC remains questionable. Nevertheless, several studies tentatively associate the circulation of anti-β-HPV antibodies and SCC [56]. For instance, seroconversion seems to increase with age [57, 58] and may be associated with an increased risk of SCC [59, 60]. Although there is no clear evidence that β-HPVs contribute directly to the development of SCCs (with the exception of EV), a recent prospective study showed that the detection of viral DNA, particularly β- and γ-HPV in the oral cavity, is associated with an increased risk of head and neck cancers, particularly with oral cavity cancers [61].

Moreover, this study identified transcripts of TVV in two OSCCs (CE02 and CE03), including one case co-infected with HPV16 (CE03). TV, a flagellated protozoan parasite, is the main non-viral agent responsible for sexually transmitted infections with more than 276 million estimated cases, 90% of which occur in resource-limited countries [62]. In high-grade lesions (HSIL) or cervical cancer, TV co-infects vaginal cells with HPV, but cannot be considered as an HPV cofactor in the genesis of precancerous lesions and their progression to malignancy [63]. However, its role in prostate cancer is more controversial, although it may be associated with a higher risk of high-grade or metastatic prostate cancer [64]. TV can induce the production of a considerable number of different pro-inflammatory cytokines such as IL-6, IL-8, MIP 3-alpha and TvMIF, which is very similar to a human cytokine, HuMIF. All of these cytokines are involved either in carcinogenesis or associated with an unfavorable carcinogenic prognosis [65–71]. Although, to date, TV has never been found associated with OSCCs, these data argue that a chronic infection with TV in the oral cavity may, via chronic inflammation, induce cell proliferation that promotes cancer, particularly in older people.

Finally, this study of the potential infectious cause of this well-characterized series of PMDs and OSCCs, carried out using a wide range of complementary molecular techniques, did not reveal the presence of any other already known oncogenic virus, with the rare exception of HR-HPV. These results are similar to those obtained by Li *et al*. (2015) on 20 cases of OSCC investigated in NS patients [29]. However, these data contradict those in Laco *et al*. (2011), which reported a much higher HR-HPV rate (12.5% vs. 4.4%) in OSCCs in NSND patients [28]. Unfortunately, the Laco *et al*. study did not specify the amount of alcohol the patients had consumed, except that they did not suffer from chronic alcoholism. Although the role of moderate alcohol consumption remains uncertain in the development of OSCC, it has a direct or indirect effect on the epithelium of the oral cavity. Alcohol consumption can act via its main metabolite, acetaldehyde, or alter the metabolism of retinoids or increase its permeability to other carcinogenic agents [72–74]. Moreover, the epithelium of the oral cavity is weakly permissive to HPV infection. The analysis of the microenvironment of the epithelium of the oral cavity confirms the absence of specialized lymphoepithelial tissue, which is present in the oropharynx. This specialized lymphoepithelial tissue fosters the HR-HPV infection of basal keratinocytes of the reticulated crypt epithelium, which promotes the development of carcinoma in these cells [13]. Therefore, the absence of this lymphoepithelial tissue in the oral cavity can explain the very low rate of HR-HPV infection observed in our NSND patients [75]. Hence, alcohol consumption may promote HR-HPV infection of basal keratinocytes in oral mucosa and thus explain the higher rate of HR-HPV infection among drinkers than among NDs in OSCCs.

In conclusion, all the results of this study strongly suggest that HR-HPV is not involved in the development of OSCCs in NSND patients and that known oncogenic infectious agents are absent in these specific OSCCs. Any possible direct or indirect role of β-HPV and TVV in NSND cases of OSCCs remains speculative and requires further investigation.

## Supporting information

S1 Table Thermal cycling for the three PCRs. (DOCX)

S2 Table List of all primers used. (DOCX)

S3 Table *Human papillomavirus* (HPV) status according to detection method (PapilloCheck and conventional PCR) for oral squamous cell carcinoma (OSCC) cases. (DOCX)

S4 Table *Human papillomavirus* (HPV) status according to detection method (PapilloCheck and conventional PCR) for potentially malignant disorder (PMD) cases. (DOCX)

S5 Table Clinical and pathological data and *Human papillomavirus* (HPV) status of all potentially malignant disorder (PMD) cases. (DOCX)

S6 Table Clinical and pathological data and *Human papillomavirus* (HPV) status of all oral squamous cell carcinoma (OSCC) cases. (DOCX)

## Abbreviations

EV: Epidermodysplasia verruciformis
HIV: Human immunodeficiency virus
HNC: head and neck cancer
HPV: human papillomavirus
HR: high-risk
ICAReB: *Investigation clinique et accès aux ressources biologiques*
LSCC: laryngeal squamous cell carcinoma
NSND: non-smoker, non-drinker
OPSCC: oropharyngeal squamous cell carcinoma
OSCC: oral squamous cell carcinoma
PMD: potential malignant disorder
PVL: proliferative verrucous leukoplakia
SCC: squamous cell carcinoma
SD: Smoker, drinker
TV: *Trichomonas vaginalis*
TVV: *Trichomonas vaginalis virus*
