# Supplementary material for: Investigation of viral etiology in potentially malignant disorders and oral squamous cell carcinomas in non-smoking, non-drinking patients
Source: PLoS One. 2020 Apr 29;15(4):e0232138. doi: 10.1371/journal.pone.0232138 (PMC7190135; doi:10.1371/journal.pone.0232138)
Supplement: S1 Table — (DOCX) [file pone.0232138.s001.docx]

**Table S1. Thermal cycling for the three PCRs**

| GP5+/6+ | | | CP65-70 | | | CP66-69 | | |
| --- | --- | --- | --- | --- | --- | --- | --- | --- |
| Time | T°C | N. of cycles | Time | T°C | N. of cycles | Time | T°C | N. of cycles |
| 5 min | 94°C | 1 | 5 min | 94°C | 1 | 9 min | 96°C | 1 |
| 30s 30s 60s | 94°C 50°C 72°C | 40 | 60s 90s 120s | 95°C 50°C 72°C | 5 | 60s 60s 120s | 95°C 55°C 72°C | 35 |
|  |  |  | 60s 90s 120s | 95°C 55°C 72°C | 35 |  |  |  |
| 5 min | 72°C |  | 5 min | 72°C | 1 | 5 min | 72°C | 1 |
| Hold |  |  | Hold | 4°C | ∞ | Hold | 4°C | ∞ |
