# Supplementary material for: Investigation of viral etiology in potentially malignant disorders and oral squamous cell carcinomas in non-smoking, non-drinking patients
Source: PLoS One. 2020 Apr 29;15(4):e0232138. doi: 10.1371/journal.pone.0232138 (PMC7190135; doi:10.1371/journal.pone.0232138)
Supplement: S2 Table — (DOCX) [file pone.0232138.s002.docx]

**Table S2. List of all primers used**

| **Name of primer** | **Target** | **Sequence 5’-3’** |
| --- | --- | --- |
| GP5+ | Β-HPV | TTTGTTACTGTGGTAGATACTAC |
| GP6+ | Β-HPV | GAAAAATAAACTGTAAATCATATTC |
| CP65 | Β-HPV | CARGGTCAYAAYAATGGYAT |
| CP66 | Β-HPV | TGTAAAACGACGGCCAGTAATCARMTGTTTRTTACWG |
| CP69 | Β-HPV | CAGGAAACAGCTATGACCGWTAGATCWACATYCCARAA |
| CP70 | Β-HPV | AAYTTCGTCCYARAGRAWATTGRTC |
| HPV33-E6-fw | HPV33 | CATGATTTGTGCCAAGCAT |
| HPV33-E7b-rv | HPV33 | TAATTGCTCATAGCAGTATAG |
| HPV16-E6E7-fw | HPV16 | GTTTCAGGACCCACAGGAG |
| HPV16-E6E7-rv | HPV16 | TGATTACAGCTGGGTTTCTCTA |
| ACTB-fw | Actin | CATCGAGCACGGCATCGTCA |
| ACTB-rv | Actin | TAGCACAGCCTGGATAGCAAC |
| TVV1F2875 (TVV1-569-F) | TVV1 | attagcggtgtttgtgatgca |
| TVV1R3443 (TVV1-569-R) | TVV1 | ctatcttgccatcctgactc |
| TVV2F2461 (TVV2-625-F) | TVV2 | GCTTGAGCACTGCTCGCG |
| TVV2-625-R | TVV2 | TCTCTTTTGGCATCGCTT |
| TVV3F61 (TVV3-440-F) | TVV3 | AAATTAATCAACACCCTCC |
| TVV3R482 (TVV3-440-R) | TVV3 | CAGATCACTTTGTGTGTC |
| TVV4F1338 (TVV4-514-F) | TVV4 | atgccagttgctttccg |
| TVV4R1834 (TVV4-514-R) | TVV4 | TTCCCCAATAGTTATCAG |
| TVV1-810-199-F* | TVV1 | CGGCCATATTTTGTCCAGAT |
| TVV1-810-199-R* | TVV1 | GTAGCTGCCCATTCCATCTC |
| TVV1-3036-194-F* | TVV1 | GCTCCCTAAACAACCCACAA |
| TVV1-3036-194-R* | TVV1 | GCCATCCTGCTTCAAAGAAC |
| TVV2-20778-199-F* | TVV2 | AGCTGGGTACCCTGAAACCT |
| TVV2-20778-199-R* | TVV2 | GGCATATTTGTTGCCGTTCT |
| TVV2-20778-202-F* | TVV2 | GACGGTTGCCATAGTGGATT |
| TVV2-20778-202-R* | TVV2 | TATGCGGAAAAGCCGATTAC |
| TVV3-4640-202-F* | TVV3 | ACATCCTCATCGAAGCCATC |
| TVV3-4640-202-R* | TVV3 | GGTGGTTCAGCAGCAATTTT |
| TVV3-4640-193-F* | TVV3 | GCACGAGGCAAAGATTTCTC |
| TVV3-4640-193-R* | TVV3 | ATCCATGACGAACTCCAAGC |
| CP41 (TV-CP4-920-F) | *Trichomonas vaginalis* | atgttcgttcaggcacatgac |
| CP42 (TV-CP4-920-R) | *Trichomonas vaginalis* | cttgtcctgtgggatgcagg |

*designed from the data obtained in high-throughput sequencing (HTS)
