# Supplementary material for: Investigation of viral etiology in potentially malignant disorders and oral squamous cell carcinomas in non-smoking, non-drinking patients
Source: PLoS One. 2020 Apr 29;15(4):e0232138. doi: 10.1371/journal.pone.0232138 (PMC7190135; doi:10.1371/journal.pone.0232138)
Supplement: S3 Table — (DOCX) [file pone.0232138.s003.docx]

**Table S3. *Human papillomavirus* (HPV) status according to detection method (PapilloCheck and conventional PCR) for oral squamous cell carcinoma (OSCC) cases**

|  |  | **HPV status** | | | | | | | | |
| --- | --- | --- | --- | --- | --- | --- | --- | --- | --- | --- |
| **Anatomical location** | **No. sample** | **PapilloCheck** | | **GP5/GP6** | | **CP65/70** | | **CP66/69** | | **Final** |
| Gum | CE01 | Negative | | Negative | | Negative | | Negative | | Negative |
|  | CE02 | Negative | | Negative | | Negative | | Negative | | Negative |
|  | CE05 | Negative | | Negative | | Negative | | Negative | | Negative |
|  | CE09 | Negative | | Negative | | Negative | | Negative | | Negative |
|  | CE13 | Negative | | Negative | | Negative | | Negative | | Negative |
|  | CE14 | Negative | | Negative | | Negative | | 5 | | 5 |
|  | CE17 | Negative | | Negative | | Negative | | Negative | | Negative |
|  | CE22 | Negative | | Negative | | Negative | | Negative | | Negative |
|  | CE25 | Negative | | Negative | | Negative | | Negative | | Negative |
|  | CE26 | Negative | | Negative | | Negative | | 15 | | 15 |
|  | CE29 | Negative | | Negative | | Negative | | Negative | | Negative |
|  | CE30 | Negative | | Negative | | Negative | | Negative | | Negative |
|  | CE39 | Negative | | Negative | | Negative | | Negative | | Negative |
|  | CE40 | Negative | | Negative | | Negative | | Negative | | Negative |
|  | CE45 | Negative | | Negative | | Negative | | Negative | | Negative |
|  | CE51 | Negative | | Negative | | Negative | | 20 | | 20 |
|  | CE52 | Negative | | Negative | | Negative | | Negative | | Negative |
|  | CE54 | Negative | | Negative | | Negative | | Negative | | Negative |
|  | CE59 | Negative | | Negative | | Negative | | 151 | | 151 |
|  | CE61 | Negative | | Negative | | Negative | | 36 | | 36 |
|  | CE62 | Negative | | Negative | | Negative | | Negative | | DL347 |
|  | CE66 | Negative | | Negative | | Negative | | Negative | | Negative |
|  | CE72 | Negative | | Negative | | Negative | | Negative | | Negative |
|  | CE73 | Negative | | Negative | | Negative | | Negative | | Negative |
|  | CE75 | Negative | | Negative | | Negative | | 20 | | 20 |
|  | CE76 | Negative | Negative | | Negative | | 14 | | 14 | |
| Mobile part of the tongue | CE03 | 16 | Negative | | Negative | | Negative | | 16 | |
|  | CE04 | Negative | Negative | | Negative | | 100 | | 100 | |
|  | CE07 | 33 | 33 | | Negative | | 36 | | 33+36 | |
|  | CE08 | Negative | Negative | | Negative | | Negative | | Negative | |
|  | CE10 | Negative | Negative | | Negative | | Negative | | Negative | |
|  | CE11 | Negative | Negative | | Negative | | 20 | | 20 | |
|  | CE18 | Negative | Negative | | Negative | | 37 | | 37 | |
|  | CE20 | Negative | Negative | | Negative | | Negative | | Negative | |
|  | CE24 | Negative | Negative | | Negative | | 113 | | 113 | |
|  | CE28 | Negative | Negative | | Negative | | Negative | | Negative | |
|  | CE37 | Negative | Negative | | Negative | | Negative | | Negative | |
|  | CE38 | Negative | Negative | | Negative | | Negative | | Negative | |
|  | CE41 | 16 | Negative | | Negative | | Negative | | 16 | |
|  | CE42 | Negative | Negative | | Negative | | Negative | | Negative | |
|  | CE44 | Negative | Negative | | Negative | | Negative | | Negative | |
|  | CE47 | Negative | Negative | | Negative | | Negative | | Negative | |
|  | CE48 | Negative | Negative | | Negative | | Negative | | Negative | |
|  | CE49 | Negative | Negative | | Negative | | 38 | | 38 | |
|  | CE60 | Negative | Negative | | Negative | | Negative | | Negative | |
|  | CE64 | Negative | Negative | | Negative | | 113 | | 113 | |
|  | CE67 | Negative | Negative | | Negative | | 23 | | 23 | |
|  | CE68 | Negative | Negative | | Negative | | 20 | | 20 | |
|  | CE74 | Negative | Negative | | Negative | | 36 | | 36 | |
|  | CE77 | Negative | Negative | | Negative | | Negative | | Negative | |
|  | PM12 | Negative | Negative | | Negative | | Negative | | Negative | |
| Cheek mucosa | CE06 | Negative | Negative | | Negative | | 36 | | 36 | |
|  | CE12 | Negative | Negative | | Negative | | Negative | | Negative | |
|  | CE32 | Negative | Negative | | Negative | | 20 | | 20 | |
|  | CE33 | Negative | Negative | | Negative | | Negative | | Negative | |
|  | CE36 | Negative | Negative | | Negative | | 37 | | 37 | |
|  | CE43 | Negative | Negative | | Negative | | 100 | | 100 | |
|  | CE65 | Negative | Negative | | Negative | | KG80 | | KG80 | |
| Inner mucosa of lips | CE34 | Negative | Negative | | Negative | | Negative | | Negative | |
| Intermaxillary region | CE15 | Negative | Negative | | Negative | | 105 | | 105 | |
|  | CE35 | Negative | Negative | | Negative | | Negative | | Negative | |
|  | CE53 | Negative | Negative | | Negative | | Negative | | Negative | |
|  | CE71 | Negative | Negative | | Negative | | 36 | | 36 | |
| Hard palate | CE21 | Negative | Negative | | Negative | | Negative | | Negative | |
|  | CE57 | Negative | Negative | | Negative | | Negative | | Negative | |
|  | CE69 | Negative | Negative | | Negative | | Negative | | Negative | |
| Soft palate | CE16 | Negative | Negative | | Negative | | Negative | | Negative | |
| Floor of mouth | CE27 | Negative | Negative | | Negative | | 20 | | 20 | |
