# Supplementary material for: Investigation of viral etiology in potentially malignant disorders and oral squamous cell carcinomas in non-smoking, non-drinking patients
Source: PLoS One. 2020 Apr 29;15(4):e0232138. doi: 10.1371/journal.pone.0232138 (PMC7190135; doi:10.1371/journal.pone.0232138)
Supplement: S4 Table — (DOCX) [file pone.0232138.s004.docx]

**Table S4. *Human papillomavirus* (HPV) status according to detection method (PapilloCheck and conventional PCR) for potentially malignant disorder (PMD) cases**

| **Anatomical location** | **No.** | **PapilloCheck** | **GP5/GP6** | **CP65/70** | **CP66/69** | **Final** |
| --- | --- | --- | --- | --- | --- | --- |
| Inner mucosa of lips | PM07 | Negative | Negative | Negative | Negative | Negative |
| Cheek mucosa | PM02 | Negative | Negative | Negative | Negative | Negative |
| Gum | PM03 | Negative | Negative | Negative | Negative | Negative |
| Mobile part of the tongue | PM01 | Negative | Negative | Negative | 9 | 9 |
|  | PM04 | Negative | Negative | Negative | Negative | Negative |
|  | PM05 | Negative | Negative | Negative | 20 | 20 |
|  | PM06 | Negative | Negative | Negative | DL347 | DL347 |
|  | PM08 | Negative | Negative | Negative | 36 | 36 |
|  | PM09 | Negative | Negative | Negative | Negative | Negative |
|  | PM11 | Negative | Negative | Negative | Negative | Negative |
|  | PM14 | Negative | Negative | Negative | Negative | Negative |
|  | PM16 | Negative | Negative | Negative | 37 | 37 |
|  | PM17 | Negative | Negative | Negative | Negative | Negative |
|  | PM18 | Negative | Negative | Negative | Negative | Negative |
