# Supplementary material for: Investigation of viral etiology in potentially malignant disorders and oral squamous cell carcinomas in non-smoking, non-drinking patients
Source: PLoS One. 2020 Apr 29;15(4):e0232138. doi: 10.1371/journal.pone.0232138 (PMC7190135; doi:10.1371/journal.pone.0232138)
Supplement: S5 Table — (DOCX) [file pone.0232138.s005.docx]

**Table S5. Clinical and pathological data and *Human papillomavirus* (HPV) status of all potentially malignant disorder (PMD) cases**

| **Anatomical location** | **No.** | **HPV**  **status** | **Age**  **(years)** | **Sex** | **Tobacco** | **Alcohol consumption** | **Hyperplasia** | **Dysplasia Grade I** | | **Dysplasia Grade II** | **Dysplasia Grade III** | ***In situ* Carcinoma** |
| --- | --- | --- | --- | --- | --- | --- | --- | --- | --- | --- | --- | --- |
| Inner mucosa of lips | PM07 | Negative | 65 | Female | NS | 3 | X |  | |  |  |  |
| Cheek mucosa | PM02 | Negative | 51 | Female | NS | ND |  |  | |  | X | X |
| Gum | PM03 | Negative | 80 | Female | NS | ND |  |  | |  |  | X |
| Mobile part of the tongue | PM01 | 9 | 82 | Female | 1 | 3 |  |  | |  |  | X |
|  | PM04 | Negative | 70 | Female | 1 | 3 | X |  | |  |  |  |
|  | PM05 | 20 | 67 | Male | 1 | 3 |  | X | |  |  |  |
|  | PM06 | DL347 | 84 | Female | NS | ND |  |  | | X |  | X |
|  | PM08 | 36 | 73 | Female | NS | ND | X |  | | X |  |  |
|  | PM09 | Negative | 72 | Female | NS | ND |  |  | |  |  |  |
|  | PM11 | Negative | 64 | Female | 1 | 3 |  |  | |  |  | X |
|  | PM14 | Negative | 80 | Female | 2 | 3 |  | X | |  |  |  |
|  | PM16 | 37 | 83 | Female | NS | 3 |  | X | |  |  |  |
|  | PM17 | Negative | 34 | Male | 2 | 3 |  |  | |  |  | X |
|  | PM18 | Negative | 75 | Female | NS | ND |  | | X |  |  |  |

1 ⬄ having stopped smoking for at least 15 years prior, regardless of initial amount smoked

2 ⬄ consumption equal to or less than 5 packs a year

3 ⬄ moderate consumption of 10 or 20 g of alcohol per day or less for women and men, respectively, or having stopped drinking for at least 15 years prior, regardless of initial consumption level

NS: Non-smoker

ND: Non-drinker
