# Supplementary material for: Investigation of viral etiology in potentially malignant disorders and oral squamous cell carcinomas in non-smoking, non-drinking patients
Source: PLoS One. 2020 Apr 29;15(4):e0232138. doi: 10.1371/journal.pone.0232138 (PMC7190135; doi:10.1371/journal.pone.0232138)
Supplement: S6 Table — (DOCX) [file pone.0232138.s006.docx]

**Table S6. Clinical and pathological data and *Human papillomavirus* (HPV) status of all oral squamous cell carcinoma (OSCC) cases**

|  |  |  |  |  |  |  | **Potential Malignant Disorders** | | |  |  |  |  |  |
| --- | --- | --- | --- | --- | --- | --- | --- | --- | --- | --- | --- | --- | --- | --- |
| **Anatomical location** | **No. sample** | **HPV status** | **Age** | **Sex** | **Tobacco** | **Alcohol consumption** | **History** | **Location** | **Time period evolution (years)** | **Histological type** | **Cellular differentiation** | **Keratinization** | **Perineural invasion** | **Vascular embolus** |
| Gum | CE01 | Negative | 61 | Male | NS | ND | Yes |  |  | no special type | Good | Yes | Yes | No |
|  | CE02 | Negative | 79 | Male | NS | 3 | No |  |  | no special type | Good | Yes | No | No |
|  | CE05 | Negative | 64 | Female | NS | 3 | No |  |  | no special type | Good | Yes | No | No |
|  | CE09 | Negative | 71 | Male | NS | 3 | No |  |  | no special type | Moderate | Yes | No | No |
|  | CE13 | Negative | 77 | Female | NS | ND | No |  |  | no special type | Good | Yes | No | No |
|  | CE14 | 5 | 72 | Female | NS | ND | No |  |  | no special type | Good | Yes | Yes | No |
|  | CE17 | Negative | 66 | Female | NS | ND | No |  |  | verrucous squamous cell carcinoma | NA | NA | NA | NA |
|  | CE22 | Negative | 70 | Male | 1 | 3 | Yes | Other | 6 | no special type | Good | Yes | No | No |
|  | CE25 | Negative | 29 | Male | 2 | 3 | No |  |  | no special type | Good | Yes | No | No |
|  | CE26 | 15 | 68 | Female | NS | ND | No |  |  | no special type | Poor | No | No | No |
|  | CE29 | Negative | 85 | Female | NS | ND | Yes | Same | 15 | no special type | Poor | No | Yes | Yes |
|  | CE30 | Negative | 58 | Female | 1 | ND | No |  |  | no special type | Good | Yes | Yes | Yes |
|  | CE39 | Negative | 82 | Female | NS | 3 | No |  |  | no special type | Moderate |  | No | No |
|  | CE40 | Negative | 75 | Male | 1 | 3 | No |  |  | no special type | Good | Yes | Yes | No |
|  | CE45 | Negative | 57 | Male | 1 | 3 | No |  |  | no special type | Good |  | No | Yes |
|  | CE51 | 20 | 65 | Female | NS | ND | No |  |  | no special type | Moderate | Yes | Yes | No |
|  | CE52 | Negative | 70 | Female | NS | ND | No |  |  | no special type | Moderate | No | No | No |
|  | CE54 | Negative | 69 | Female | NS | ND | No |  |  | no special type | Good | Yes | Yes | Yes |
|  | CE59 | 151 | 79 | Female | NS | 3 | No |  |  | no special type | Moderate | Yes | No | No |
|  | CE61 | 36 | 83 | Female | NS | ND | No |  |  | no special type | Moderate | No | No | No |
|  | CE62 | DL347 | 89 | Female | NS | ND | No |  |  | no special type | Good | Yes | No | No |
|  | CE66 | Negative | 84 | Male | 1 | 3 | No |  |  | no special type | Good |  | No | No |
|  | CE72 | Negative | 56 | Male | NS | 3 | No |  |  | no special type | Moderate | Yes | Yes | No |
|  | CE73 | Negative | 83 | Female | NS | ND | No |  |  | no special type | Moderate | Yes | Yes | Yes |
|  | CE75 | 20 | 68 | Male | NS | ND | No |  |  | no special type | Good | Yes | No | No |
|  | CE76 | 14 | 84 | Female | NS | ND | Yes | Same | 7 | no special type | Moderate | Yes | No | No |
| Mobile part of the tongue | CE03 | 16 | 49 | Female | NS | ND | No |  |  | no special type | Good | Yes | NA | NA |
|  | CE04 | 100 | 83 | Female | NS | 3 | No |  |  | no special type | Moderate | Yes | Yes | Yes |
|  | CE07 | 33+36 | 53 | Male | NS | ND | No |  |  | no special type | Good | Yes | No | No |
|  | CE08 | Negative | 37 | Male | NS | ND | No |  |  | no special type | Good | Yes | Yes | No |
|  | CE10 | Negative | 69 | Male | 1 | 3 | Yes | Other | 20 | no special type | Good | Yes | Yes | Yes |
|  | CE11 | 20 | 83 | Female | 2 | 3 | No |  |  | no special type | Good | Yes | No | No |
|  | CE18 | 37 | 48 | Male | NS | 3 | No |  |  | no special type | Moderate | Yes | Yes | No |
|  | CE20 | Negative | 69 | Male | 1 | 3 | Yes | Same | 7 | no special type | Good | Yes | No | No |
|  | CE24 | 113 | 82 | Female | NS | ND | No |  |  | no special type | Poor | No | Yes | No |
|  | CE28 | Negative | 55 | Male | NS | 3 | Yes | Same | 5 | no special type | Moderate | Yes | Yes | Yes |
|  | CE37 | Negative | 36 | Male | NS | 3 | No |  |  | no special type | Good | Yes | No | No |
|  | CE38 | Negative | 40 | Female | NS | 3 | No |  |  | no special type | Good |  | No | No |
|  | CE41 | 16 | 61 | Female | 1 | 3 | Yes | Same | 1 | no special type | Good | Yes | No | No |
|  | CE42 | Negative | 87 | Female | 2 | ND | No |  |  | no special type | Moderate | Yes | Yes | Yes |
|  | CE44 | Negative | 85 | Male | 1 | 3 |  |  |  | no special type | Moderate | Yes | Yes | No |
|  | CE47 | Negative | 73 | Female | NS | 3 | Yes | Same | 4 | no special type | Good | Yes | No | No |
|  | CE48 | Negative | 77 | Male | 1 | ND | No |  |  | no special type | Moderate | Yes | Yes | No |
|  | CE49 | 38 | 85 | Male | 1 | ND | No |  |  | no special type | Good |  | Yes | No |
|  | CE60 | Negative | 82 | Female | NS | 3 | No |  |  | no special type | Moderate | No | No | No |
|  | CE64 | 113 | 53 | Male | NS | 3 | Yes | Same | 7 | no special type | Moderate | No | No | No |
|  | CE67 | 23 | 66 | Female | NS | ND | No |  |  | no special type | Good | Yes | No | No |
|  | CE68 | 20 | 67 | Male | 1 | 3 | Yes | Same | 1 | no special type | Good | Yes | NA | No |
|  | CE74 | 36 | 76 | Female | NS | ND | No |  |  | no special type | Good |  | No | No |
|  | CE77 | Negative | 70 | Female | NS | ND | Yes |  |  | no special type | Moderate | Yes | Yes | No |
|  | PM12 | Negative | 36 | Male | 2 | 3 | Yes | Same | 1 | no special type | Moderate | Yes | No | No |
| Cheek mucosa | CE06 | 36 | 64 | Female | NS | 3 | No |  |  | no special type | Good | Yes | No | No |
|  | CE12 | Negative | 86 | Male | 2 | ND | No |  |  | verrucous squamous cell carcinoma | Good | No | No | No |
|  | CE32 | 20 | 92 | Female | NS | 3 | No |  |  | no special type | Good | Yes | Yes | Yes |
|  | CE33 | Negative | 80 | Male | 1 | 3 | Yes | Other | 12 | no special type | Good | Yes | No | No |
|  | CE36 | 37 | 50 | Male | NS | 3 | No |  |  | no special type | Good |  | No | No |
|  | CE43 | 100 | 77 | Male | NS | ND | No |  |  | no special type | Poor |  | No | No |
|  | CE65 | KG80 | 89 | Male | 1 | 3 | No |  |  | no special type | Moderate |  | No | No |
| Inner mucosa of lips | CE34 | Negative | 63 | Female | NS | ND | No |  |  | no special type | Good |  | Yes | No |
| Intermaxillary region | CE15 | 105 | 80 | Male | 1 | 3 | No |  |  | no special type | Moderate |  | Yes | No |
|  | CE35 | Negative | 81 | Male | NS | ND | No |  |  | no special type | Good | Yes | Yes | No |
|  | CE53 | Negative | 71 | Male | NS | ND | No |  |  | epidermoid carcinoma with fusiform cells | Poor | Yes | No | No |
|  | CE71 | 36 | 61 | Female | NS | ND | No |  |  | no special type | Moderate | Yes | Yes | Yes |
| Hard palate | CE21 | Negative | 72 | Male | 1 | 3 | No |  |  | no special type | Good | Yes | No | No |
|  | CE57 | Negative | 63 | Female | NS | ND | No |  |  | no special type | Moderate | No | Yes | Yes |
|  | CE69 | Negative | 83 | Female | NS | ND | No |  |  | no special type | Moderate | Yes | No | No |
| Soft palate | CE16 | Negative | 35 | Female | NS | ND | No |  |  | no special type | Good | Yes | NA | NA |
| Floor of mouth | CE27 | 20 | 52 | Male | 2 | ND | No |  |  | no special type | Good | Yes | No | Yes |

1 ⬄ having stopped smoking for at least 15 years prior, regardless of initial amount smoked

2 ⬄ consumption equal to or less than 5 packs a year

3 ⬄ moderate consumption of 10 or 20 g per day or less for women and men, respectively, or having stopped drinking for at least 15 years prior, regardless of initial consumption

NS: Non-smoker

ND: Non-drinker
